# Case Report: Endoscopic-guided electrochemotherapy combined with metronomic chemotherapy for the treatment of nasal tumors in dogs

**DOI:** 10.3389/fvets.2026.1756592

**Published:** 2026-02-11

**Authors:** Giulia Maggi, Alfredo Dentini, Giuseppe Giovannini, Chiara Paloni, Davide De Lorenzi, Maria Chiara Marchesi

**Affiliations:** 1 Department of Medicine Veterinary, University of Perugia, Perugia, Italy; 2 Anicura Tyrus Veterinary Clinic, Terni, Italy; 3 San Marco Veterinary Clinic and Laboratory, Padova, Italy

**Keywords:** dog, electrochemotherapy, endoscopy, nasal tumor, oncology

## Abstract

Electrochemotherapy is a local anticancer treatment used for selected cutaneous tumors, but its application in veterinary medicine for nasal neoplasms is only rarely reported. Two dogs with endonasal tumors, ineligible for radiotherapy, underwent endoscopic-guided electrochemotherapy (ECT). Each cycle included three sessions spaced 3 weeks apart. Under general anesthesia and endoscopic guidance, bleomycin (20,000 IU/m^2^) was administered intravenously, followed by bipolar electroporation via a new single needle electrode. Palliative metronomic chemotherapy (piroxicam, thalidomide, and cyclophosphamide) was also administered as an adjuvant to the local treatment. Both dogs showed reduced tumor size and resolution of clinical signs after the third ECT session. Only mild intraoperative bleeding was observed. Follow-up CT and endoscopy confirmed significant volumetric tumor reduction and improved nasal airflow. Endoscopic-guided ECT combined with metronomic chemotherapy appears to be a promising palliative approach for canine endonasal tumors, providing tumor cytoreduction and improvement of clinical signs. Further studies with larger sample sizes and longer follow-up periods are needed to confirm the safety and efficacy of this approach, either alone or in combination with other surgical or medical treatments.

## 1 Introduction

Electrochemotherapy (ECT) is a local treatment used for the cytoreduction of certain solid tumors, particularly those localized in cutaneous and subcutaneous tissues. The principle of ECT is based on the increased permeabilization of cell membranes through *electroporation*, which enhances the penetration of chemotherapeutic cytotoxic drugs into the cells. Electroporation is achieved by delivering electric pulses via electrodes applied to the target tissue. High-voltage electric pulses alter the cell membrane potential, resulting in the rearrangement of the lipid bilayer and the formation of pores. These pores allow the passage of medium and large molecules across the membrane without specific carrier (1, 2).

Tumors of the nasal cavity has mostly epithelial origins, and represents approximately 1–2% of all the neoplasms that occur in dogs (3). These tumors primarily have a local impact, with progressive invasion of surrounding tissues and bone structures, and a lower rate of metastasis. When metastasis occurs, it typically involves regional lymphnodes and the lungs (4). Radiotherapy using high-energy megavoltage (MeV) equipment as the sole therapy is the gold standard treatment for dogs with nasal tumors. Other treatments reported include sole surgical therapies (such as conventional rhinotomy and endoscopic debridement), chemotherapy, and cryosurgery, as well as combinations of traditional or mini-invasive surgery with radiation (5–7). The safe use of ECT for the treatment of nasal cavity tumors has been reported in the literature (8, 9). In a study by Maglietti et al. (8), the procedure was performed using a single-needle electrode (SiNE) inserted directly into the affected nasal cavity without image or endoscopic guidance. The study demonstrated the feasibility of ECT for the treatment of nasal tumors, with variable therapeutic responses and minimal adverse effects.

In this case report, we describe two dogs with endoscopic, computed tomographic (CT), and histologic findings consistent with nasal tumors underwent endoscopic-guided ECT. A novel electrode, specifically designed for the treatment of deep neoplasms, was used to induce electroporation of the nasal tumors under endoscopic guidance, aiming to maximize treatment efficacy in combination with palliative metronomic chemotherapy. The owners were informed that radiotherapy is the first-line treatment for this type of tumor; however, due to financial constraints, this option was not pursued. Informed consent was obtained from all subjects involved in the study. The aim of this study is to retrospectively describe and evaluate a novel electrode for endoscopic-guided ECT in the nasal cavity, focusing on its technical feasibility, potential complications, and clinical outcomes in dogs with nasal tumors treated in combination with chemotherapy.

## 2 Cases description

### 2.1 Case 1

**Figure 1:**
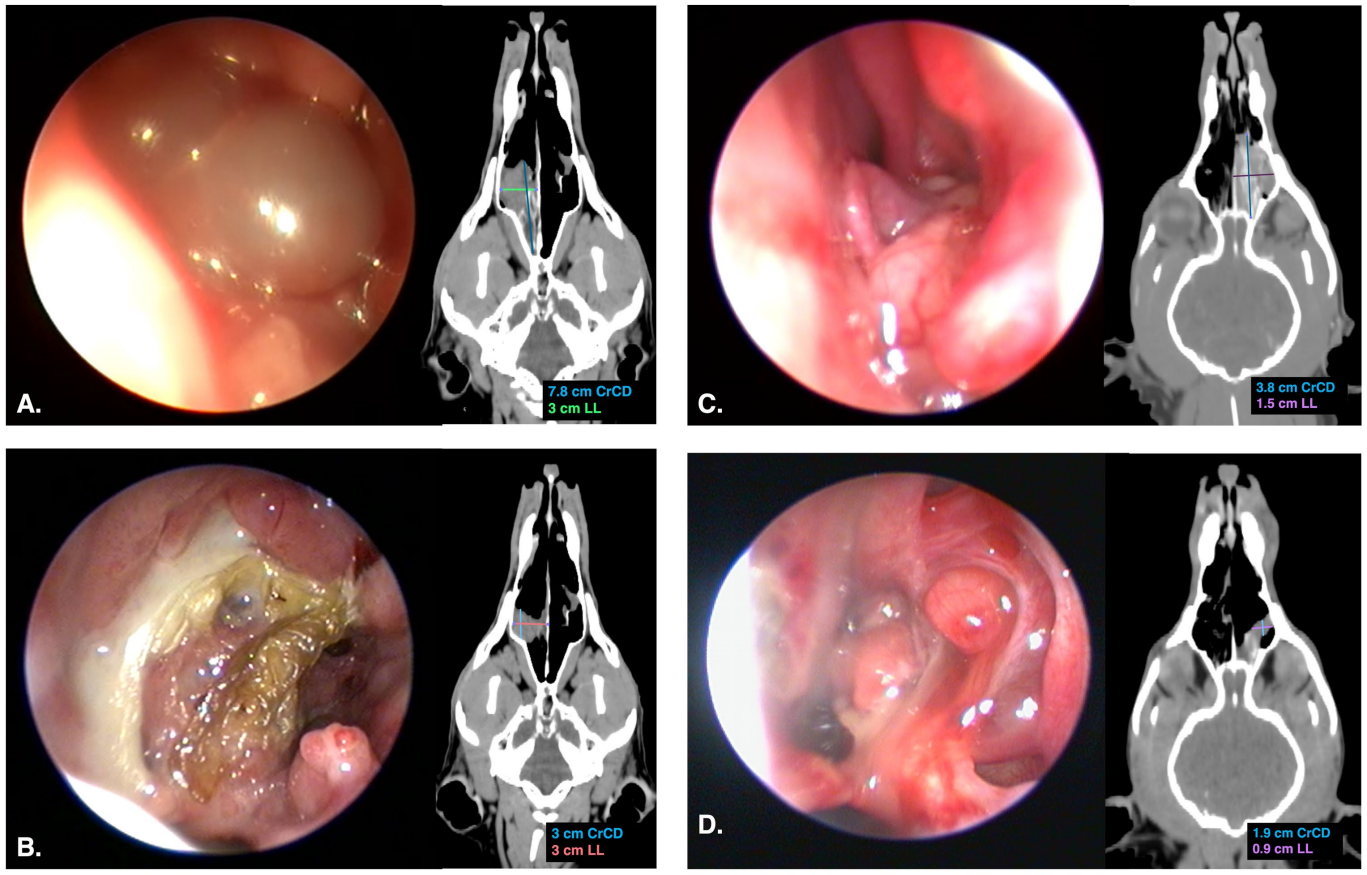
Endoscopic and dorsal CT images of nasal tumors obtained during first ECT sessions and at follow-up; maximum diameters of the mass were measured on dorsal CT planes. **(A)** Endoscopic and dorsal CT images acquired before the first ECT session in Case 1; endoscopic examination reveals a rosy-colored mass with a smooth surface, occupying approximately half of the nasal cavity and completely filling the nasal meatus; CT scan revealed a contrast-enhancing, hypoattenuating endonasal mass occupying and nearly occluding the aboral third of the right nasal cavity. **(B)** Endoscopic and CT images acquired at the 1.5-month follow-up in Case 1; at endoscopic examination, the neoformation was confined mainly to the middle meatus and partially to the dorsal meatus, in the context of turbinate atrophy, mucopurulent exudate, and an eschar surrounding the surface of the neoformation; CT scan demonstrated a marked reduction in the CrCD and LL dimensions of the mass. **(C)** Endoscopic and CT images acquired before the first ECT session in Case 2; endoscopic examination revealed a rosy-colored, vascularized neoformation with an irregular surface, completely filling the middle nasal meatus and partially the dorsal meatus; CT scan revealed a space-occupying endonasal neoformation localized to the middle-aboral third of the left nasal cavity, resulting in obliteration of the entire caudal endoluminal segment. **(D)** Endoscopic and CT images acquired at the 1.5-month follow-up in Case 2; endoscopic examination revealed remodeling of the original lesion into two distinct small nodular formations, with exposure of atrophic nasal turbinates; CT scan revealed a reduction of the mass, predominantly in the CrCD direction and, to a lesser extent, in the LL direction.

A 13-year-old intact male Siberian Husky was presented to the Oncology Service of Anicura Tyrus Veterinary Clinic with a prior diagnosis of undifferentiated nasal carcinoma, made on July 24, 2024. The tumor was classified as stage II (T3N0M0) according to the World Health Organization (WHO) TNM classification system, causing deformation of the frontonasal profile, stertorous breathing, nasal stridor, and occasional epistaxis (10). Physical examination revealed previously described abnormal respiratory sounds, absence of airflow from the right nasal cavity, and deformation of the frontonasal profile with pain elicited on palpation of the deformed area. A head CT scan was performed prior to each endoscopic-guided ECT to determine the dimensions of the endonasal mass. The tumor volume was calculated using the formula *V = abcπ/6* accordance with the Standard Operating Procedures for ECT published by Tozon et al. (1). Head CT scan revealed a contrast-enhancing, hypoattenuating endonasal mass occupying and nearly occluding the aboral third of the right nasal cavity, associated with osteolysis of the right nasal bone and nasal septum (Figure 1A). Anterior rhinoscopy was performed using a 2.7 mm × 18 cm and 1.9 mm × 18 cm rigid telescopes (Telescope K Storz 64018BS, Karl-Storz-Endoscopy, Tuttlingen, Germany) with a halogen light source. Nasopharyngeal endoscopic examination was carried out using a 5.2 mm × 85 cm flexible endoscopic (Fiberscope K Storz 11278AK, Karl-Storz-Endoscopy). Rhinoscopy revealed a rosy-colored mass with a smooth surface and firm, elastic consistency, occupying approximately half of the nasal cavity and completely filling the nasal meatus (Figure 1A).

**Figure 2:**
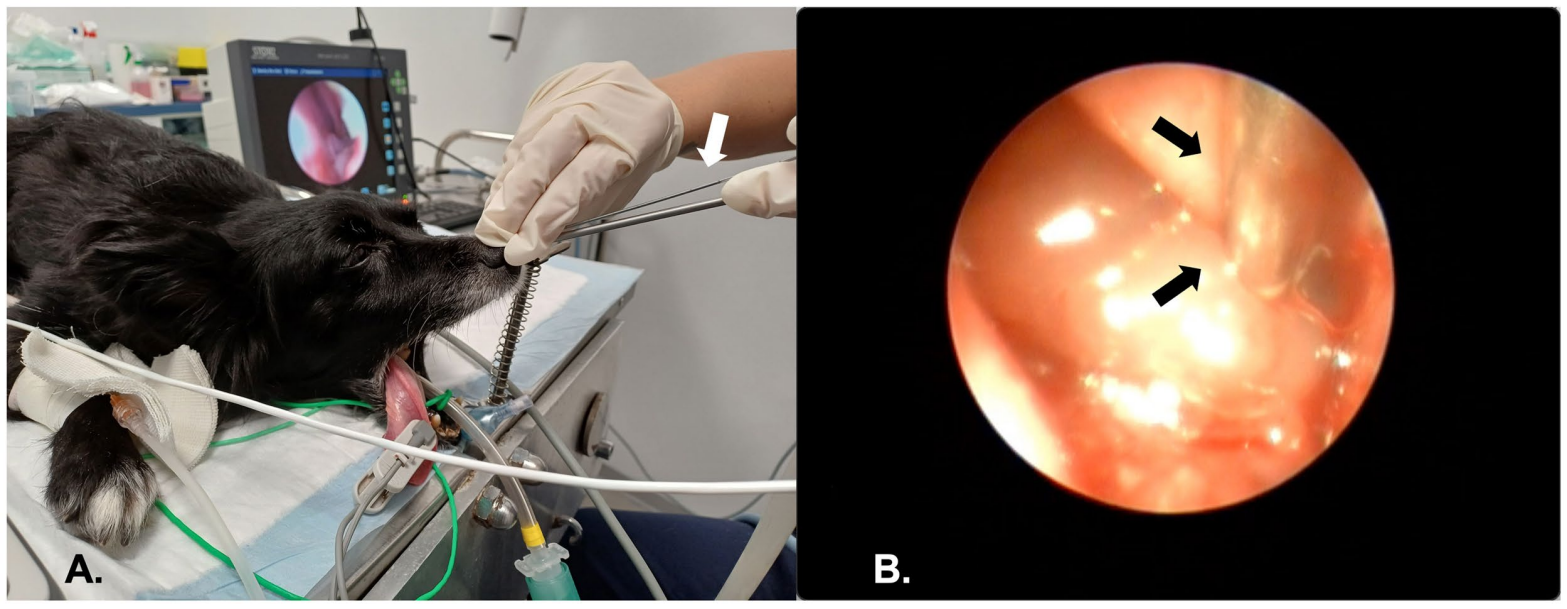
The figure shows endoscopic-guided ECT for the treatment of a nasal tumor in a dog. The arrows indicate the bipolar single needle electrode. **(A)** Dog positioning during anterior rhinoscopy. All procedures were performed with the dog in sternal recumbency, with the jaw held open using a gag. Inspection was conducted with a rigid telescope, while ECT was performed by inserting a needle electrode coaxially with the endoscope. **(B)** Endoscopic view of ECT; the needle electrode was inserted into the mass under endoscopic guidance.

Each cycle of ECT consisted of three administrations, performed at 3-week intervals (11). Endoscopic-guided ECT was performed under general anesthesia in all dogs. After endoscopic visualizing the intranasal neoformation, 20,000 IU/m^2^ of bleomycin were administered intravenously over 60 s (11, 12). Eight minutes later according to the Operating Procedures of the ECT published by Tozon et al. (1), ECT was performed using a bipolar single needle electrode (VGD 21 Gauge KOSMO, IGEA S.p. A, Carpi, Italy) inserted coaxially with the endoscope (Figure 2A) and connected to a square-wave generator (CLINIPORATOR^®^ VITAE, IGEA S.p. A, Carpi, Italy). The electrical pulses were emitted from the needle tip, radiating over a surface area of 1 cm^2^. The electric pulses consisted of four trains of pulses with an amplitude of 800 V, a pulse length of 100 μs, and a frequency of 5,000 Hz. Under direct visualization, the needle was inserted into the mass and penetrated deeply along its full length (Figure 2B), as determined by the CT scan dimensions. Electrical pulses were then delivered, with the needle retracted by 1 cm after each discharge. Depending on the height of the mass, the procedure was repeated by reinserting the needle 1 cm lower into the lesion, ensuring the entire height of the mass was treated. This approach ensured complete electroporation of the neoformation. The procedure was completed within 20 min of administering bleomycin. A medical therapy consisting of piroxicam at 0.3 mg/kg once daily, thalidomide at 4 mg/kg once daily, and cyclophosphamide at 12.5 mg/m^2^ once daily, was administered starting from the first ECT and throughout the entire follow-up period. First session of endoscopic-guided ECT was performed on September 25, 2024. Only one intra-operative complication was recorded during the second session of endoscopic-guided ECT, which involved mild bleeding. This was controlled by the application of cold water, adrenaline, and nasal tampons in the nasal cavity.

**Figure 3:**
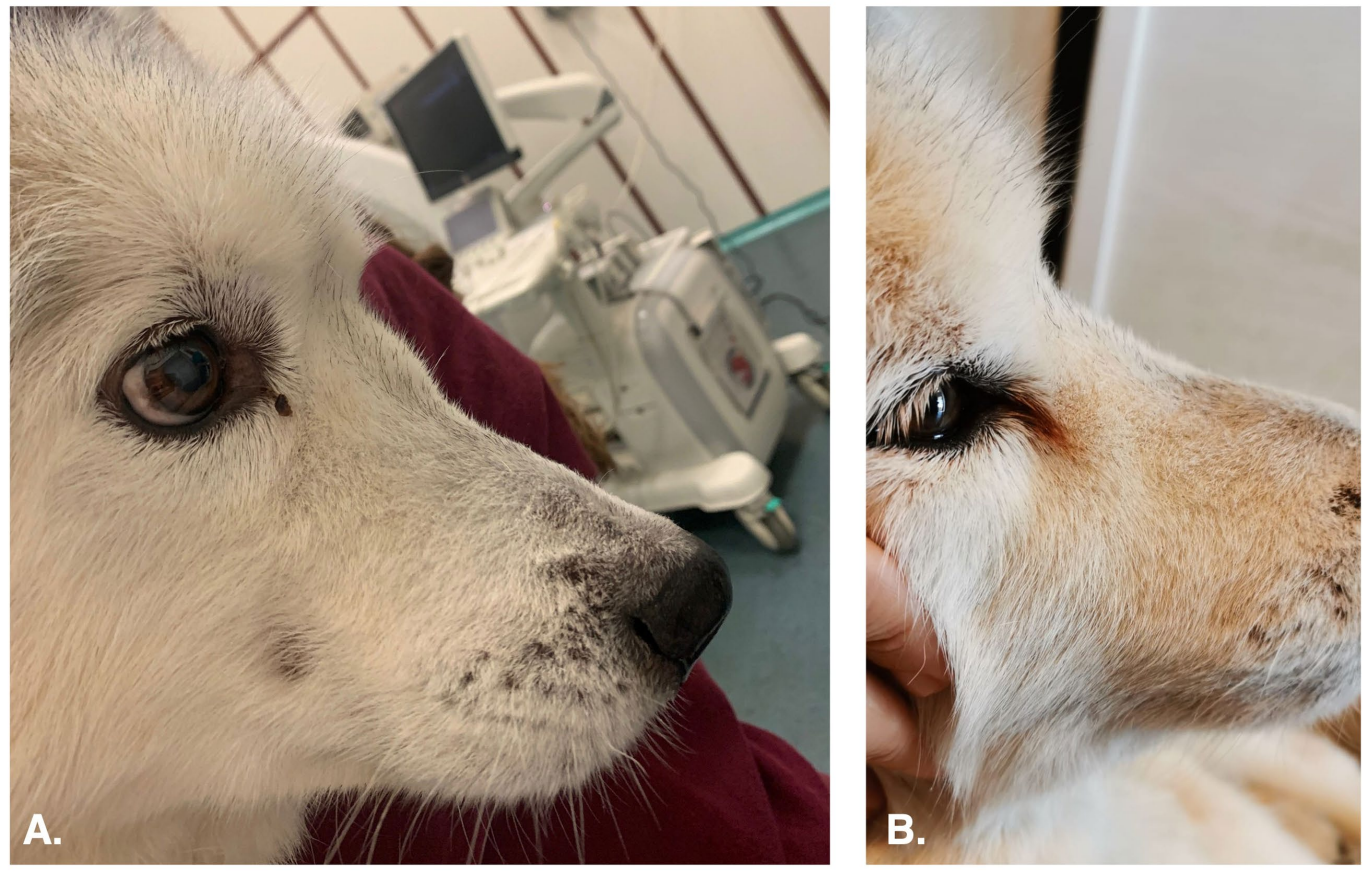
The figure shows the appearance of the fronto-nasal profile in a dog before and after the first session of ECT. **(A)** Lateral view of the fronto-nasal profile of a dog affected by undifferentiated nasal carcinoma before endoscopic-guided ECT. The profile appeared deformed. **(B)** Lateral views of the fronto-nasal profile of the same dog after the first session of ECT; the deformation had disappeared.

The nasal carcinoma showed a reduction in size, as revealed by head CT scan and endoscopic examination prior to local treatment, with significant shrinkage observed particularly after the first and third ECT sessions. The dimensions and volume of the nasal carcinoma, measured through head CT scan before each ECT session and at follow-up, are presented in Table 1. At the completion of the three ECT sessions, the nasal carcinoma showed an 81% reduction in tumor volume compared with baseline measurements. According to the WHO’s Response Evaluation Criteria, this outcome was classified as a partial response (PR) (13). Endoscopic examination showed a significant and progressive increase in the available space within the nasal cavity, with gradual retraction of the mass observed at each session and during follow-up (1.5 months). After the first ECT session, the mass involved the dorsal and middle meatus and only partially the common meatus, whereas by the second ECT session it was confined mainly to the middle meatus and partially to the dorsal meatus. The reduction of the mass also revealed rarefaction of the nasal turbinates. Mild edema, hyperemia, and the presence of white mucous exudate on the nasal mucosa were observed during each local treatment. Additionally, an eschar was noted during follow-up (Figure 1B). Occasional episodes of epistaxis persisted throughout the observation period, while respiratory sounds, such as stertorous breathing and nasal stridor, disappeared after the third ECT session. Moreover, the reduction of the nasal mass was evident by the disappearance of the fronto-nasal deformation (Figure 3) after the first endoscopic-guided ECT. The dog died spontaneously from unknown causes, apparently unrelated to the neoplastic pathology, 99 days after the first ECT session and 162 days after diagnosis.

**Table 1:** Dimensions of the nasal carcinoma measured through head CT scans before each endoscopicguided electrochemotherapy (ECT) session, and during follow-up.

Case	I° endoscopic-guided ECT session CrCD × VD × LL (cm); Vol. (cm^3^)	II° endoscopic-guided ECT session CrCD × VD × LL (cm); Vol. (cm^3^)	III° endoscopic-guided ECT session CrCD × VD × LL (cm); Vol. (cm^3^)	1.5 months follow-up CrCD × VD × LL (cm); Vol. (cm^3^)
Case 1	7.8 × 4.1 × 3 cm; 50.2 cm^3^	5 × 4 × 2.5 cm; 26.2 cm^3^	5 × 3.7 × 2.1 cm; 20.3 cm^3^	3 × 2 × 3 cm; 9.4 cm^3^
Case 2	3.8 × 2.1 × 1.5 cm; 6.3 cm^3^	2.5 × 2.1 × 1.2 cm; 3.3 cm^3^	1.9 × 2.1 × 1.1 cm; 2.3 cm^3^	1.9 × 2.1 × 0.9 cm; 1.88 cm^3^

CrCD, cranio-caudal; ECT, electrochemotherapy; LL, latero-lateral; VD, ventro-dorsal; Vol., volume.

### 2.2 Case 2

A 12-year-old neutered female mongrel was presented to the Oncology Service of Anicura Tyrus Veterinary Clinic with a prior diagnosis of well-differentiated transitional carcinoma made on 14 September, 2024. The tumor was classified as stage I (T2N0M0) according to the WHO TNM classification system (10). Physical examination revealed no abnormalities, except for absent airflow from the left nasal cavity and abnormal respiratory sounds. Clinical signs related to the tumor included nasal stridor, nasal and reverse sneezing, and occasional unilateral hemorrhagic discharge. A head CT scan and endoscopy were performed prior to each endoscopic-guided ECT, as previously performed for Case 1. Head CT scans revealed a space occupying endonasal neoformation localized to the middle-aboral third of the left nasal cavity, resulting in obliteration of the entire caudal endoluminal segment (Figure 1C). Ventral extension showed incipient invasion of the left choana, which remained normally aerated. Partial invasion of the left maxillary recess by the lesion was confirmed, with no evidence of paranasal osteolysis. Endoscopic examination of the left nasal cavity revealed a rosy-colored, vascularized neoformation with an irregular surface and elastic consistency, occupying approximately the caudal third of the cavity and completely filling the middle nasal meatus e partially the dorsal meatus (Figure 1C). The mass extended into the left choana, as revealed by rhino-pharyngeal examination.

As with the previous dog (Case 1), endoscopic-guided ECT was proposed as an alternative treatment. First session of endoscopic-guided ECT was performed on November 19, 2024. A medical therapy consisting of piroxicam at 0.3 mg/kg every 48 h, thalidomide at 4 mg/kg every 48 h, and cyclophosphamide at 12.5 mg/m^2^ every 48 h, were administered starting from the first ECT session and continued throughout the entire follow-up period. Only one immediate post-operative complication occurred during the first session of endoscopic-guided ECT, consisting of mild bleeding. This was managed with the application of cold water, adrenaline, and nasal tampons in the nasal cavity. After the first endoscopic-guided ECT, the dog experienced increased nasal and reverse sneezing.

The nasal carcinoma showed a reduction in size during the course of endoscopic-guided ECT, with significant shrinkage observed particularly after the first and third ECT sessions. The dimensions and volume of the nasal carcinoma measured before each ECT session and at the follow-up are presented in Table 1. At the end of the three ECT sessions, the nasal carcinoma was reduced 70.2% compared to its initial dimensions. According to the WHO’s Response Evaluation Criteria, this outcome was classified as a partial response (PR) (13). A significant increase in the space within the nasal cavity and a reduction in the neoformation were evidenced by TC head scan and endoscopic examination after first session and during follow-up control. The reduction of the mass occurred mainly in the craniocaudal (CrCD) direction and, to a lesser extent, in the latero-lateral (LL) direction. Endoscopic evaluation demonstrated a progressive reduction in tumor size, as indicated by an increased patency of the middle and dorsal meatus. At the second ECT session, a moderate amount of mucopurulent material was present within the nasal cavity. During the third ECT session, an eschar associated with focal hemorrhagic suffusion was observed. At follow-up, exposure of atrophic nasal turbinates was evident. Moreover, remodeling of the original lesion was noted, with the mass appearing as two distinct small nodular formations, a finding confirmed by both CT and endoscopic examinations (Figure 1D). Respiratory sounds, including nasal wheezing, and all other clinical signs resolved after the second ECT session. At 131 days after the first ECT session, the dog presented with a recurrence of epistaxis. By day 173, stertor had also developed as a new clinical sign. However, the owner declined further diagnostic evaluations, including CT and endoscopy, to assess potential tumor regrowth. Euthanasia was performed due to severe clinical signs related to the tumors (obstructive respiratory distress), occurring 208 days after the first ECT session and 274 days after diagnosis.

## 3 Discussion

This report describes the treatment of canine nasal tumors using a novel electrode specifically designed for deep electroporation under endoscopic guidance, combined with adjuvant metronomic chemotherapy, which proved effective in achieving tumor cytoreduction in two dogs.

Electrochemotherapy (ECT) is a locoregional antitumor treatment increasingly used for cytoreduction of solid tumors. Its mechanism of action is based on reversible electroporation, a biophysical process in which short, high-intensity electric pulses transiently increase cell membrane permeability. These electric pulses are generated by a pulse generator and delivered to the target tissue through specifically designed electrodes (1). Electroporation facilitates the intracellular uptake of poorly permeant cytotoxic agents, which otherwise have limited ability to cross the cell membrane. Exposure of cells to pulsed electric fields induces the formation of transient aqueous pores (*electropores*) in the lipid bilayer, allowing diffusion of ions, water, and chemotherapeutic molecules. Additionally, electric fields promote transient clustering of transmembrane proteins, forming pseudopores that further enhance molecular transport across the membrane (1, 2). Bleomycin and cisplatin are the chemotherapeutic agents most commonly used in clinical ECT protocols and may be administered either intravenously or intratumorally. The increased intracellular drug accumulation markedly enhances local cytotoxicity, allowing effective tumor control with lower drug doses and reduced systemic toxicity. Beyond direct cytotoxicity, ECT exerts additional antitumor effects (1, 2). These include a vascular-disrupting effect, characterized initially by a reversible vascular lock with reduced tumor blood flow, followed by endothelial cell damage in small vessels, leading to prolonged ischemia (14, 15). Furthermore, tumor cell death induced by ECT promotes the release of tumor-associated antigens, contributing to immune system activation and a secondary antitumor immune response (1). Clinical oncology studies have demonstrated that ECT is effective in the treatment of cutaneous and subcutaneous tumors in both humans and animals, with a curative response observed in approximately 80% of cases (1, 16). Some studies have shown that, in certain cases, ECT can be an equivalent or even superior treatment compared to surgery for specific tumors (1, 17). In other cases, ECT is used with palliative intent, including for tumors located in areas that have been previously irradiated or surgically treated (11, 16).

Only a few previous studies have reported the use of ECT for the management of nasal cavity neoplasm (8, 9). Suzuki et al. (9) documented, for the first time, the technical application of multiple needles into the nasal cavity and the use of conductive gel via a surgical approach to achieve an appropriate electric field. To reduce the invasiveness of the procedure, Maglietti et al. (8) performed a single ECT treatment using a SiNE inserted into the nasal cavity. In this study, electroporation was performed without endoscopic guidance; only CT or magnetic resonance (MR) imaging was used to guide the procedure and prevent inadvertent penetration through the cribriform plate. In our cases, we took advantage of the low invasiveness of the single-electrode technique, one of the main benefits described by Maglietti et al. (8) through the use of a novel bipolar single electrode. However, to further improve the efficacy and safety of the procedure, we employed not only CT guidance but also direct visualization of the neoplasm through endoscopic guidance. This approach allowed us to increase the treated area, addressing one of the main limitations of the previously described technique, the inability to cover the entire tumor effectively, while also reducing the risk of electroporating healthy nasal tissue (7). As reported in recent studies, bleomycin was administered intravenously at an increased dose intensity (20,000 IU/m^2^) to enhance treatment efficacy (11, 12). To achieve better local control of the mass and prevent regrowth, three treatment sessions were performed at three-week intervals, following the protocol previously described by Moretti et al. (11). In fact, although ECT can be effective with a single treatment, in cases of partial tumor response it can be repeated multiple times, achieving equal or even improved effectiveness (1, 18). Finally, as recently suggested by other authors, metronomic chemotherapy was used to enhance the local effect of ECT (7).

Radiotherapy, alone or in combination with surgery, are the gold standard for treating nasal tumors; however, complete remission is rarely achieved, and most tumors only decrease in size (19). The limited availability of radiotherapy facilities and the high cost of treatment make it inaccessible for many pet owners; moreover, tumors that recur after radiotherapy often exhibit reduced sensitivity to radiation (7, 20). Many animals that experience recurrence of the neoplasm, or that are unable to undergo radiotherapy due to economic or other reasons, require alternative therapeutic options. Currently, the only available palliative treatment strategies are surgery and endoscopic debulking (7, 20). Rhinotomy is an invasive treatment that has not demonstrated a significant increase in life expectancy and is associated with a high rate of morbidity (7, 20). Endoscopic debulking with laser is a minimally invasive alternative that can be used alone or in combination with radiotherapy. The advantage of this treatment lies in the ability to ablate the neoplasm without the need for surgical access, resulting in shorter hospitalization times and faster recovery for the animals. However, multiple treatment sessions may be necessary and a complete remotion of the mass is rarely obtained (20). Endoscopic-guided ECT, as described in this report, shares similarities with endoscopic debulking; however, debulking procedures can be lengthy, lasting up to 72 min (7), whereas ECT sessions were completed in no more than 20 min, consistent with the active window of the cytotoxic chemotherapeutic agent in the bloodstream.

In our cases report, a nasal carcinoma and a transitional carcinoma were treated using endoscopic-guided ECT, resulting in a reduction in tumor size for both cases. The cytoreduction outcomes were confirmed through head CT scans and rhinoscopic examinations performed during treatment, as well as during follow-up. Head CT scans showed a decrease in tumor size and volume. Rhinoscopic examination revealed an increase in nasal cavity space, rarefaction of the nasal conchae, and a reduction in the nasal neoformation. According to Maglietti et al. (8), in which 64% of animals achieved a PR, the objective response observed in both of our cases was comparable. Considering that the antitumor effectiveness of ECT depends on tumor size, with smaller tumors responding better than larger ones, our preliminary results, in agreement with previous reports, indicate that nasal tumor reduction can also be effectively achieved through endonasal ECT (1). Although large prospective studies are limited, a recent case report describes successful partial remission of a canine nasal carcinoma treated with a long-term metronomic combination of chlorambucil and prednisolone (21). Although the exact contribution of chemotherapy as an adjuvant to local ECT treatment is unknown, additional evidence from metronomic protocols in dogs with nasal tumors demonstrates antitumor activity and clinical benefit (7), supporting the potential role of chemotherapy in the management of rhino-nasal neoplasms.

Only mild bleeding was observed as an intraoperative complication during endoscopic-guided ECT. Blood loss was managed with the application of cold water, adrenaline, and nasal tampons in the nasal cavity, along with the parenteral administration of tranexamic acid. Similar complications have been previously reported for other minimally invasive palliative treatments for nasal tumors, including endoscopic-guided procedures (20). During electroporation, a temporary vasoconstriction occurs, reducing blood flow by up to 80%. This effect helps to retain chemotherapeutic drugs within the tumor, enhancing their cytotoxic potential, while simultaneously limiting nutrient supply to the tumor and reducing the risk of hemorrhage (8, 14, 15). In the literature, severe hemorrhage has been reported as a complication during endoscopic laser debulking, obstructing the view and preventing completion of the procedure (20). In our cases, bleeding did not prevent electroporation of the mass. The blood was effectively aspirated through the endoscope’s working channel, allowing all procedures to be completed successfully.

In a study evaluating the clinical outcomes of dogs with nasal tumors treated with radiotherapy followed by endoscopic laser debulking, only 44.4% of the animals showed a significant reduction in clinical signs. Moreover, many animals were euthanized due to the severity of clinical signs that compromised their quality of life (20). In contrast, a recent study by Bottero et al. (7) identified endoscopic debridement as a valuable palliative treatment for animals with nasal tumors, due to its positive impact on quality of life. Since complete remission of endonasal tumors is not achievable with currently available treatments, the primary therapeutic goal (particularly in veterinary medicine) is the alleviation of tumor-related symptoms, while minimizing side effects as much as possible. In this case report, each dog presented with clinical signs consistent with a nasal tumor, including nasal and reverse sneezing, nasal discharge, and abnormal respiratory sounds. In the first dog, treatment led to an improvement in clinical symptoms, with only occasional hemorrhagic discharge persisting throughout the study period. In the second case, ECT resulted in a complete resolution of clinical signs for several months, with a recurrence of epistaxis occurring 131 days (≅ 4 months) after the initiation of the ECT protocol. Considering that most animals either succumb to their condition or are euthanized due to severe clinical signs, this approach provides a palliative option that improves quality of life in affected animals.

Several radiological side effects may occur following radiotherapy, particularly inflammation or infection (bacterial or fungal) of adjacent regions to the irradiated site (e.g., eyes, mouth, nasal cavity, brain etc.). In the most severe cases, these complications can become chronic and may necessitate the placement of a feeding tube for enteral nutrition (8, 20). Previous applications of ECT for the treatment of nasal neoplasms reported only one side effect (nasal passage inflammation) which was managed with a one-week course of corticosteroid therapy (8). In our cases, although moderate signs of local inflammation (e.g., edema, hyperemia, presence of eschar) were observed during endoscopic inspection throughout the ECT sessions, no clinical signs of inflammation were reported, and no additional treatment was required. This favorable outcome may be attributed to the concurrent administration of piroxicam throughout the study period, used as an adjuvant to chemotherapy. At the same time, the selective electroporation of only the affected tissue (ensured by endoscopic guidance) may contribute to a reduction in local inflammation.

The overall mean survival time for dogs treated with radiotherapy ranges from 8 to 19 months, whereas the mean survival time after surgery or chemotherapy without irradiation, or in the absence of treatment, is 3–6 months (19, 22, 23). The mean survival time for dogs treated with a combination of radiation and laser debulking was 18 months (20). Similar results were reported in the only study that used ECT for the treatment of nasal tumors, where the overall mean survival time was 16.86 months (8). In our experience, one dog died spontaneously 99 days after the initiation of treatment with endoscopic-guided ECT, due to unknown causes that appeared unrelated to the tumor pathology. The total survival time for this dog, calculated from the time of diagnosis, was 162 days (≅ 5.5 months). Another dog was euthanized due to recurrence of severe clinical signs related to the tumor at 208 days from the first ECT session, with a total survival time from diagnosis of 274 days (≅ 9 months). Unfortunately, the available data are limited and do not allow for a reliable comparison of survival time after endoscopic-guided ECT with other treatments reported in the literature. However, both our findings and those of Maglietti et al. (8) are promising and underscore the need for further studies involving larger cohorts of dogs and extended follow-up periods.

The principal limitations of this case report are its retrospective nature and the small number of treated animals, which preclude statistical analysis and definitive conclusions. In addition, the combined use of local ECT and metronomic chemotherapy does not allow the specific contribution of ECT to be precisely determined, as the observed clinical response may have been influenced by the concomitant medical therapy. Considering the deep localization of nasal tumors, which often precludes curative surgical excision with clear margins, and the high cost of radiotherapy, endonasal ECT using a specifically designed electrode, in combination with adjuvant metronomic chemotherapy, may represent an effective palliative treatment option. In the treated animals, this approach resulted in tumor size reduction and improvement of clinical symptoms. Further studies involving larger cohorts, as well as investigations assessing the efficacy of endonasal ECT alone and in combination with other treatments, particularly endoscopic debulking and metronomic chemotherapy, are needed. Such studies would support the development of a standardized and reproducible protocol, similar to those established for other solid cutaneous and subcutaneous tumors.

## Data Availability

The raw data supporting the conclusions of this article will be made available by the authors, without undue reservation.
